# PSI: A Comprehensive and Integrative Approach for Accurate Plant Subcellular Localization Prediction

**DOI:** 10.1371/journal.pone.0075826

**Published:** 2013-10-23

**Authors:** Lili Liu, Zijun Zhang, Qian Mei, Ming Chen

**Affiliations:** College of Life Sciences, Zhejiang University, Hangzhou, China; Lawrence Berkeley National Laboratory, United States of America

## Abstract

Predicting the subcellular localization of proteins conquers the major drawbacks of high-throughput localization experiments that are costly and time-consuming. However, current subcellular localization predictors are limited in scope and accuracy. In particular, most predictors perform well on certain locations or with certain data sets while poorly on others. Here, we present PSI, a novel high accuracy web server for plant subcellular localization prediction. PSI derives the wisdom of multiple specialized predictors via a joint-approach of group decision making strategy and machine learning methods to give an integrated best result. The overall accuracy obtained (up to 93.4%) was higher than best individual (CELLO) by ∼10.7%. The precision of each predicable subcellular location (more than 80%) far exceeds that of the individual predictors. It can also deal with multi-localization proteins. PSI is expected to be a powerful tool in protein location engineering as well as in plant sciences, while the strategy employed could be applied to other integrative problems. A user-friendly web server, PSI, has been developed for free access at http://bis.zju.edu.cn/psi/.

## Introduction

Discovering the subcellular localization of proteins provides important clues for revealing their function and aids in understanding their interactions with other biomolecules at the cellular level [1], [2].The eukaryotic cell is particularly highly organized, in which various protein functions and biological processes are associated with specialized subcellular localizations. The localization of proteins with known function can either aid in annotation of newly discovered or sequence-inferred proteins or unravel how and under what cellular compartments intricate pathways regulate biological process. Some powerful experimental approaches, such as proteomics and microscopic detection of tagged or labeled proteins have been proposed [3]. However, these are invariably expensive and time-consuming. With the development of high throughput sequencing technology, a wealth of protein sequence data has been accumulated. It is now highly desirable to develop computational methods for protein subcellular localization prediction based on amino acid sequence information.

Over recent years, various prediction methods and tools have been introduced [4]–[23]. Most of these tools employ the machine learning method to predict protein localization via learning localization specific sequence features with proteins of known localizations. Signal peptide and sequence similarity are widely used sequence features to identify a protein's location within the cell by predictors such as mitoProt [24], TargetP [8], Predotar [7] and PASUB [25] etc. Newly-developed methods consider annotation information including function domain and motifs [21], gene ontology terms [22] and textual information [23], [24]. Some predictors combine several features. For example, N-peptide composition and physio-chemical properties were used together by ESLpred [26]. Likewise, MultiLoc exploits amino acid composition, N-terminal targeting sequences and motifs [27].

Although much progress has been made in predicting plant subcellular localization, current predictors remain limited in their scope and accuracy. To the best of our knowledge, only five predictors can cover all of the following ten locations: (1) cytosol, (2) endoplasmic reticulum, (3) extracellular space, (4) Golgi, (5) membrane, (6) mitochondrion, (7) nucleus, (8) peroxisome, (9) plastid, and (10) vacuole. Others can discriminate plant proteins among only few location sites. For instance, TargetP [8] can cover three locations while iPSORT can only capture two sites. Commonly, any given predictor may perform well on certain locations or data sets but poorly on others. Often predictions by different prediction tools disagree with each other. This probably results from the employment of different algorithms, a differing choice of training set, or the different sequence features they exploit etc. Previous publications have reported that an integration of various prediction methods may outperform the individual methods [28]. Some meta-servers for protein subcellular location prediction were proposed using the voting method or machine learning method [29]. However, the group voting method ignores non-linear effects between predictor results and the neural network tends to sacrifice accuracy of minor cellular compartments to reach an optimized overall prediction result. Meanwhile, the performance of the neural network method can be improved with addition of prior knowledge. Our main inspiration was to unite the two integration methods (using group-voting results as neural network inputs) to conquer more challenging tasks.

Here, we present PSI, a highly accuracy web server for plant subcellular localization prediction. This represents the first attempt to employ the combination of group voting, the group decision making strategy, and the machine learning method artificial neural network, to give an integrated best result. PSI conquers the limitation in scope, accuracy and location prediction bias consist in most other individual prediction tools. It can cover all of the following ten locations: (1) cytosol, (2) endoplasmic reticulum, (3) extracellular space, (4) Golgi, (5) membrane, (6) mitochondrion, (7) nucleus, (8) peroxisome, (9) plastid, and (10) vacuole.

## Results

### Prediction bias

**Figure 1:**
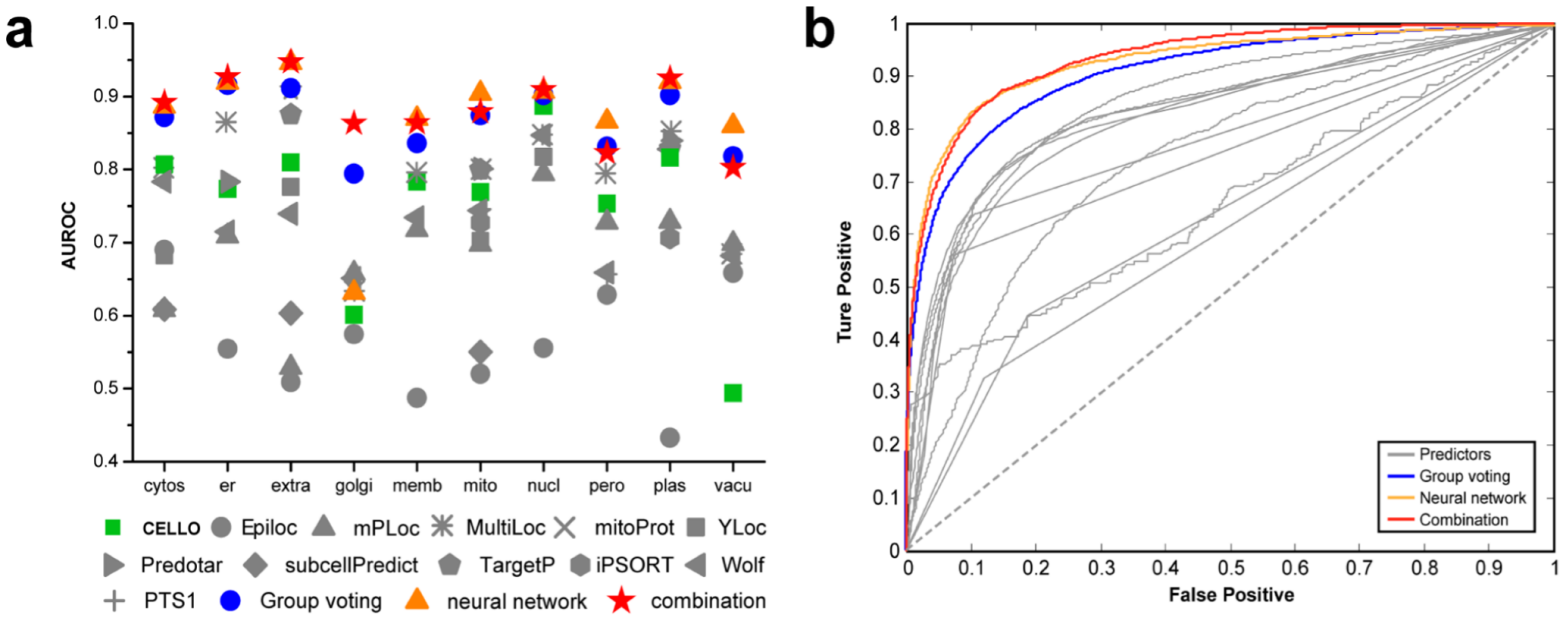
Performance evaluation. (a) AUROC values for individual subcellular locations reflected a varied prediction power of different predictors and on different location sites. Three integration strategies including group-voting (blue sphere), neural network (saffron triangle) and combination (red stars) improved prediction power significantly for all subcellular locations. (b) Overall ROC-curves illustrated the significant elevation of prediction power after integration. Grey lines denote the performance of the individual predictors. Blue line describes the performance of the group voting method; the saffron line shows the accuracy of the neural network method and the red line characters the performance of the combination method.

From the assessment of each individual predictor, a prediction bias was reflected (Figure 1). For each certain predictor the performance at a different subcellular location varied. This may be due to differences in the employment of algorithms, choice of training set, or sequence features they exploit etc. For example the best individual predictor with highest overall AUROC value, CELLO, actually performed rather poorly on prediction for the vacuole. Meanwhile, some subcellular locations, such as the Golgi apparatus, lacked accurate predictors. Successful integration strategy takes advantages of the differing complementary strengths of each of the location predictors. Thus, whilst the predictors' performance varied, gains in accuracy are realized through accumulation and through the non-linear relationships for locations where all predictors performed poorly.

### Community integration using group-voting

#### Ranking system

Predictor results for each protein sequence were first separated into ten nodes for ten subcellular compartments with their corresponding confidence score. The ranking system for the separated compartment-specific nodes was established according to confidence score among the all prediction confidence scores given by that predictor. For each node, an identifier was given and the normalized score was calculated according to the function described in the method. The ranking results and normalized scores for individual predictors were presented in Table S3. True positive (TP) nodes and false positive (FP) nodes are denoted by labeling the judge column 1 or 0 corresponding to the gold standard. Consequently the normalized score of the TP nodes are higher than that of the FP nodes' after calculation according to the method. The T-test indicated a significant difference between the mean values of the two distributions. Figure S1 shows the TP vs. FP frequency-distribution diagrams. Rank band was 0.01 ranging from 0 to 1. Meanwhile the distribution for best individual predictor, CELLO, and integration of three predictors, CELLO, WoLF PSORT and MultiLoc was depicted in the same way. The mean difference between the two peaks was observable and significant in the full-integrated community while evaluation of AUROC reflected a better prediction power when integration increased from 1 to 3 and from 3 to 11.

#### Performance evaluation

**Figure 2:**
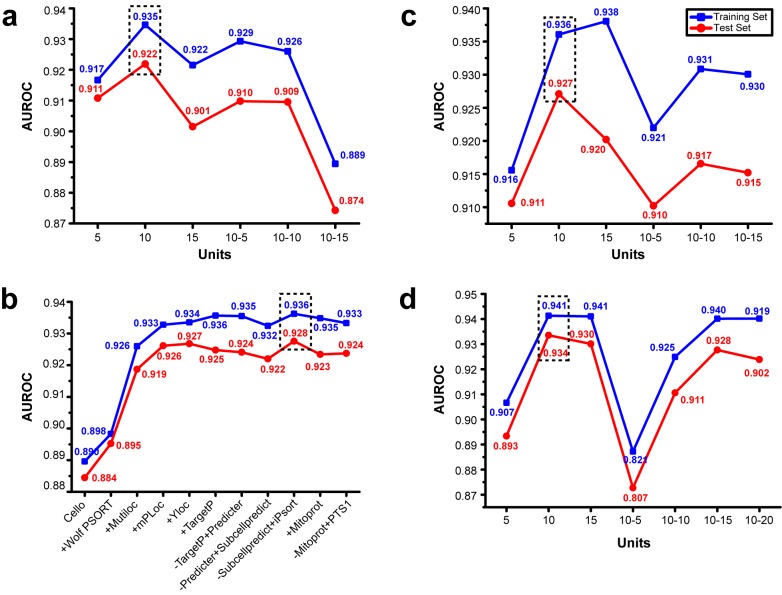
Determination of the model topological structure. (a) Performance under different model structures with full data as input. Blue lines denote the training set and red lines denote the test set. Peak for prediction was on 10 neurons, 1 hidden layer. (b) Stepwise-selection performance was evaluated by AUROC. Obvious enhancement took place in step 2, 3 and 4. Peak was in step 9, with best community consisting of cello, Wolf PSORT, MultiLoc, mPloc, YLoc and iPsort. (c) With selected community of predictors, model topological structure was determined using the same method in (a). Peak was on 10 neurons, 1 hidden layer. (d) Model structure evaluation for combination of group-voting and neural network. Results from group-voting were taken as input for neural network. Peak was on 10 neurons, 1 hidden layer. The best results are boxed in dash.

Using the integrated ranking system, an average ranked list was given with an average score ranging from 0 to 1 representing an increased confidence of the prediction. Evaluation was performed against the ROC curve (Figure 2). From the AUROC values, it was verified that the community integration of predictors outperformed each individual predictor, both overall and on individual subcellular location (Figure 2).

### Community integration using artificial neural-network

#### Topological structure determination

To determine the best neural network structure, all predictor results were formed sequentially as inputs for network training. Structure of 1, 2 hidden layers with 5, 10, 15 neurons respectively in each layer was trained and the prediction power was evaluated by the test set via AUROC. Note that as the neuron number increases in addition to the increasing complexity of the model, the learning power also always increases while the prediction power has a peak, due to the over-fitting phenomenon. Since the neural network performed differently every time and gave local optimized results by chance, each structure was trained 10 times and the best 3 ones were picked out to give a statistical illustration of optimized results. The structure with the best prediction power was proven to be that of one hidden layer of ten neurons (Figure 2a).

#### Stepwise-selection of predictors

With the determined structure of the neural network, stepwise selection was performed to determine the best community of predictors. Predictors were added according to their prediction power (AUROC value) from high to low. A predictor was conserved in the community if the prediction evaluation values (AUROC) for test set increased after addition; otherwise it was removed in the next step. The detailed stepwise-selection performance is shown in Figure 2b. Consequently the best community for prediction consists of six predictors: (1) CELLO, (2) WoLF PSORT, (3) MutiLoc, (4) mPLoc, (5) YLoc, and (6) iPSORT. With the selection of feature vectors from the above six predictors, the model which achieved the higher accuracy was revealed by AUROC while the complexity of the model could be reduced to avoid over-fitting, Different topological structures were trained and evaluated again using the same method and the selected community of predictors. As a result, one hidden layer of ten neurons was found to be the best structure with the best prediction power (Figure 2c).

#### Performance evaluation

Prediction results on test sets were given via a neural network using 1 hidden layer with 10 neurons, based on data from community of six selected predictors. Since the neural network performed variably each time, the model with highest overall performance, as evaluated by AUROC was selected from among large amounts of trained models. The overall performance evaluation illustrated that the neural network method outperformed the group-voting method as a whole (Figure 1). However on specified subcellular locations, the neural network prediction performed rather poorly, e.g. on Golgi apparatus. This is because unbiasedness cannot be guaranteed, due to the black-box feature of neural networks. Therefore, a neural network usually sacrifices the accuracy of minor cellular compartments to reach the optimized overall prediction results.

### Community integration outperforms individual predictors

With comprehensive comparison among the group-voting method, artificial neural network method and each individual predictor's results, a clear enhancement of prediction power evaluated by AUROC was discovered. Figure 1 shows that community integration outperforms all individual predictors in overall prediction accuracy, where the improvement of prediction power by the neural network is more significant than that of group-voting. However, group-voting guaranteed a lift of accuracy on each specified subcellular location while the neural network sacrificed accuracy of minor cellular compartments, such as Golgi apparatus, to reach the optimized overall prediction results. This is because neural network itself is a black-box algorithm and unbiased property is not guaranteed. Group voting has been mathematically proved to be unbiased improving the prediction power as long as a better than random predictor performance is added. In all, our results verified that the integration of predictors outperforms each individual predictor and community integration was required for accuracy.

### Wisdom of group-voting and neural network combination for subcellular localization prediction

**Figure 3:**
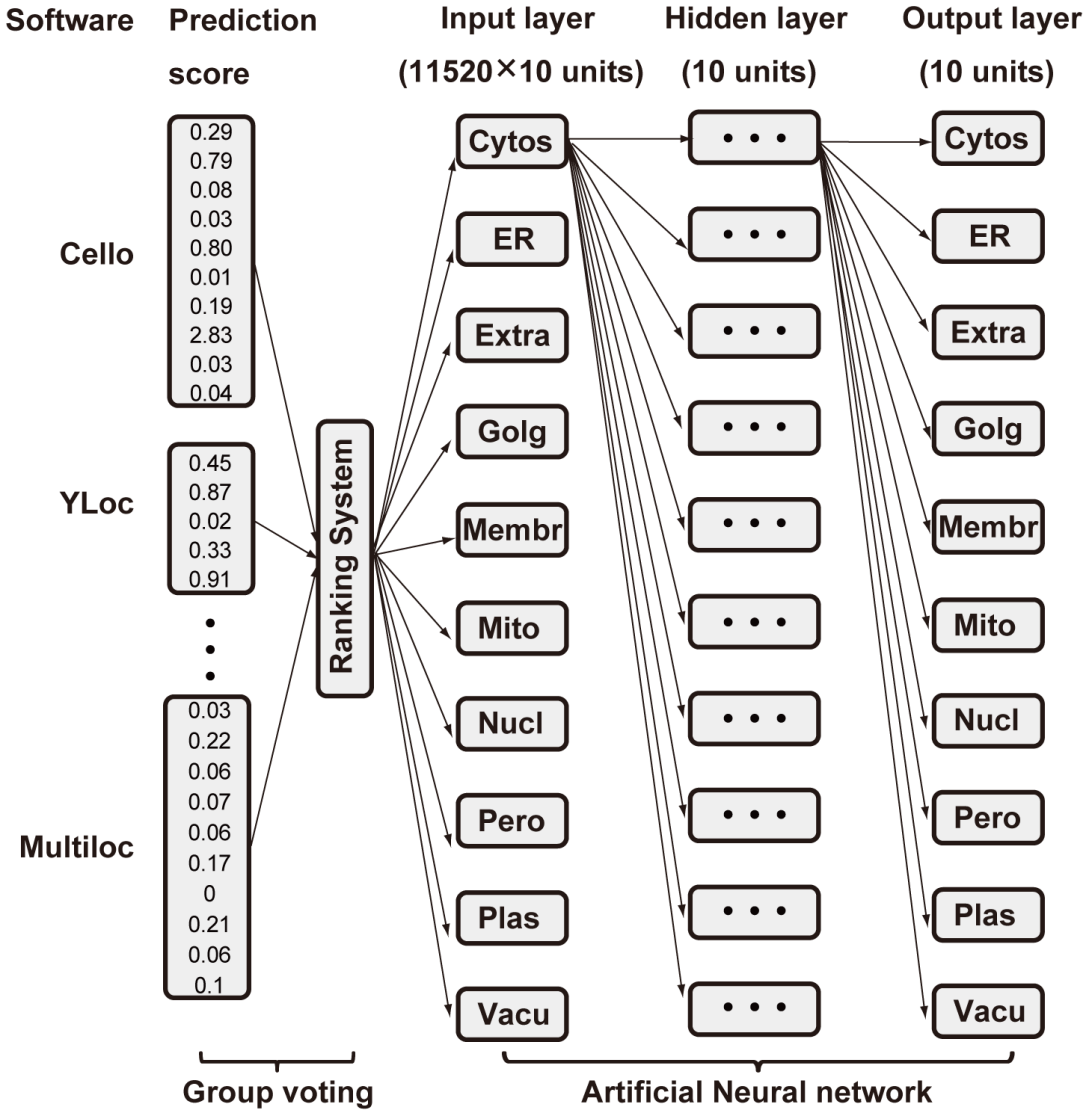
Model for combination of group voting and neural network. Raw prediction results from 11 predictors first entered ranking system to give prediction for each subcellular location of elevated-accuracy. Then neural network took group-voting output as input for further calculation and adjustment. Final prediction results were given through neural network.

We must consider that the group voting method ignores the non-linear effects between predictor results where the neural network method tends to sacrifice the accuracy of minor cellular compartments, like Golgi apparatus, to reach the optimized overall prediction results. Both integration strategies have their strengths and limitations. Meanwhile, the neural network method can improve its performance with addition of prior knowledge. The strategy for integration of the two methodologies is shown in Figure 3.

Using group-voting results as neural network inputs, the topological structure was then determined. As a result, one hidden layer of ten neurons was the best structure with the most prediction power (Figure 2d). The overall performance for the combination improved to AUROC∼0.934, which outperformed either group-voting (AUROC∼0.906) or neural network (AUROC∼0.928) alone (Figure 1). Despite the overall better performance, the combination model is better but not significantly different to the results from the neural network alone. However, the neural network itself is a black-box algorithm where unbiased property is not guaranteed, which results in rather poor prediction performance on specified subcellular locations e.g. on Golgi apparatus. The combination performed well with an AUROC≥0.8 for each predictable subcellular location, which was a better result than that of group-voting on most sites, while it maintained a high prediction power for minor subcellular locations that were lost in the neural network method.

### Webserver implementation

The PSI-predictor took the input protein sequence in a FASTA format. Both pasted sequences and files were acceptable for input, with the pasted items limited to 20 sequences and with a file limitation of 500 kb. The format was checked before submission.

Using the input sequences, predictor results were retrieved and formatted. A webpage of visualization, to reflect the predictor status, was shown after submission. Whenever a predictor's result was responded to, the indication of ‘ready to use’ would appear and be hyperlinked to the individual result in a readable format provided by the predictor itself.

After all predictor results were provided, the integration algorithms were employed. Integrated prediction results were given when all collection and integrations were complete. These are illustrated in the result page with scores ranging from 0 to 1 for each of the subcellular locations, i.e. extracellular, cytosol, membrane, endoplasmic reticulum, mitochondria, Golgi apparatus, plastid, nuclear, vacuole and peroxisome. A higher score represents a higher confidence of the protein's existence in certain cellular compartments whilst the P-values, as the statistical inference in the brackets, show the confidence of the results. In the result page, different P-value thresholds could be selected to filter the results shown on the screen while in the downloaded file all original data was reserved in a format that is easily imported into XLS files. The webserver is accessible at http://bis.zju.edu.cn/psi/

### Comparison between PSI and other individual predictors

According to the cross validation with area under the ROC curve (AUROC) [32], the area under the precision vs. recall curve (AUPR) [32], F-score [33] and Matthews Correlation Coefficient (MCC) [33] (the detail results were shown in Table S4), overall performance for PSI improved to AUROC∼0.934, AUPR∼0.644, F-score∼0.568 and MCC∼0.54, which outperformed all individual predictors. As determined by ANOVA (the results were presented in Table S2), the performance of PSI is significantly better than CELLO, mPLoc, MultiLoc, WoLF PSORT, Predotar, subcellPredict, Yloc at p<0.05 level; there is no significant difference between TargetP and PSI, though TargetP only covers three locations (extracellular space, mitochondrion and plastid). In addition, other individual predictors (iPSORT, mitoProt and PTS1) can only discriminate one or two locations, which results in the N/A ANOVA results. In conclusion, PSI significantly improved the plant's subcellular location prediction power.

### The applicability of PSI in other plants

To explore the applicability of PSI in other plants, PSI has been used to predict protein entries (drawn from Swiss-Prot database) in Rice, Soybean, Spinach, Tabacco, Tomato and Barley (Table S5). The results indicate that PSI can be used for more than the two species discussed and would be potentially suited for plant-wide use.

## Discussion

### Why we need employ the combination of group-voting and neural network?

The purpose of any integration strategy is to make good use of strengths of each component whilst offsetting their weaknesses, in order to give an overall more accurate prediction than any individual part. Prediction bias is the foundation as well as the difficulty to overcome for any successful integration strategy. These biases may come from different input features that the predictors seek, different algorithms employed, and the different training sets upon which the algorithm was built. For the specified subcellular localization problem, prediction bias can be classified into three sources, namely, complementary effects, accumulation effects and non-linear effects.

#### Complementary effects

Complementary effects were considered where the subcellular location had both accurate predictors and poor predictors. Based on prediction bias, different predictors compensate for each other's poorly predicted locations. For example, the individual predictor MultiLoc gave the good predictions on average, but performed relatively poor for the Golgi apparatus and nucleus, which could be compensated for when other predictors' results, such as CELLO or WoLF PSORT, were added.

#### Accumulation effects

The accumulation effect is especially important for the subcellular locations where none of the predictors gave accurate predictions. Some subcellular locations, such as the vacuole and peroxisome, thus far lack any accurate predictors. All the individual predictors collected performed unsatisfactorily (Figure 2). SVD analysis confirmed the independency of each individual predictor that is the specific rank of an edge given by a predictor has no dependency on the same edge's rank from any other predictors. Due to the statistical independency of predictor results, a more correct prediction result was obtained when more predictors were integrated. As the number of predictors integrated increases, the prediction accuracy of poorly predicted locations from all individual predictors will finally rise to a satisfying level. Here, to give an unbiased accumulation and integration of prediction, the group-voting method was employed.

#### Non-linear effects

In formatting and integrating prediction results, non-linear effects were noticed to be considerably imported for predictor integration. For example, mitoProt gives a probability score that a certain protein exists in the mitochondria. However, according to mitoProt's performance in the training set, a relatively high score was given to mitochondria as well as to the plastid. Similar relationships of scoring were supposed to exist in both intra- and inter-predictors. Such intrinsic correlations are important sources of information that could be integrated to give a better prediction, based on which artificial neural-network method was introduced.

Furthermore, an integrative strategy can resolve conflicting predictions made by elemental predictors which frustrate experimenters and researchers in that it is hard to make a concrete decision based on those predicted values. Group voting, as a statistical technique for prediction integration, has been rigidly proven to improve prediction power whenever an independent predictor that performs better than random is added. Our results further confirmed the effect of group voting by showing that integration of predictors performed better than individual predictors both on each of the subcellular locations and overall.

On the other hand, the neuron network took the non-linear effects into consideration and turned out to be better than that of the pure arithmetic average scores given by the group voting. A theoretically optimized model of artificial neuron network could give the same results as the combination of neural network and group voting together where group voting results are used as the input. In this, the group voting results actually act as prior knowledge to help the neural network find the optimized parameters.

Nevertheless, the combination of group-voting and neural network is currently the best approach that provides the most reliable prediction results. It takes all the effects proposed above (complementary effects, accumulation effects and non-linear effects) into consideration and takes advantage of both group-voting and neural network methods.

### Influence of experimental data as input on result output

As only the experimental data was taken as gold standard, the training set data is actually biased due to the imbalance of studies for different cellular compartments. Well-studied compartments, such as mitochondria or chloroplast, tend to contain more diverse patterns while some minor compartments, such as the vacuole and Golgi apparatus, contain fewer patterns. This phenomenon results in a more sensitive output in the well-studied compartments while giving a more specific output in the less-studied compartments.

### Towards more powerful predictions

No matter how delicate and skillful the integration strategy develops, the true threshold for highly accurate predictions always lies in the deficiency of the individual predictors. Some of subcellular locations are currently benefiting from a large amount of study, such as for plastid and nucleus for which predictors of high confidence have been readily developed. However some subcellular locations, like Golgi apparatus and vacuoles, lack accurate predictors. Although integration of predictors generally improved prediction accuracy for these locations e.g. from AUROC 0.68 to 0.80 after applying the algorithm for the vacuole, the accuracy remains far from satisfactory. Herein we call for predictors specified for the ‘minor’ subcellular locations, which are equally important for the portrait of the whole biological systems picture.

Currently only the static spatial picture is depicted via prediction while, in biological systems, proteins are modified and transported to different subcellular compartments from time to time. Taking regulations of protein levels, such as protein modifications and protein-protein interactions into account, we could expect a more powerful predictor that responds to predictions of spatial as well as temporal distribution when given a protein sequence. This acquires a higher emphasis upon protein targeting, and certainly makes more sense in systems biology.

Subcellular localization of proteins is considered to be important for protein function. Based on predictions of high accuracy, function analysis, such as clustering and protein family distribution analysis, can be conducted. Data mining from a confident prediction of protein targeting helps to gain insight in biological pathway process and evolution, which is the ultimate goal of subcellular localization prediction.

## Materials and Methods

### Experimental data and gold standard

Protein with curated locations from SUBA3 [30] and PPDB [31] were used as our datasets, including 16009 protein sequences located in ten sites: (1) cytosol (cytos), (2) endoplasmic reticulum (ER), (3) extracellular space (extra), (4) Golgi apparatus (Golgi), (5) membrane (membr), (6) mitochondria (mito), (7) nucleus (nucl), (8) peroxisome (pero), (9) plastid (plast) and (10) vacuole (vacu) from Arabidopsis and maize in order to build a meta-predictor specific to plants. The number of protein sequence within each location site was more than 100. In the SUBA3 database only the locations derived by experiment (including Location Green Fluorescent Protein, Location Mass Spec and Protein-protein Interaction) were used as gold standards while the consensus locations by several predictors were not included. Consequently, among the datasets, one-fifth was randomly picked out as the test set and the rest as the training set. Protein subcellular locations verified via experiments were taken as the gold standard. Note that for a protein sequence, subcellular locations which were not verified should be considered as potentially existent rather than non-existent. Thus it is reasonable to evaluate only true positive and false positive rates, which will be further illustrated in “Assessment of subcellular localization predictors”.

### Assessment of subcellular localization predictors

Totally of 22 currently published subcellular predictors were collected. Among the community 11 predictors were chosen for dataset prediction, which either provide a standalone version, batch prediction or response of a single prediction within 1 min. Other predictors were either off-shelf or the response was too slow to predict mass data. A detailed summary of currently available predictors is given in Table S1.

**Figure 4:**
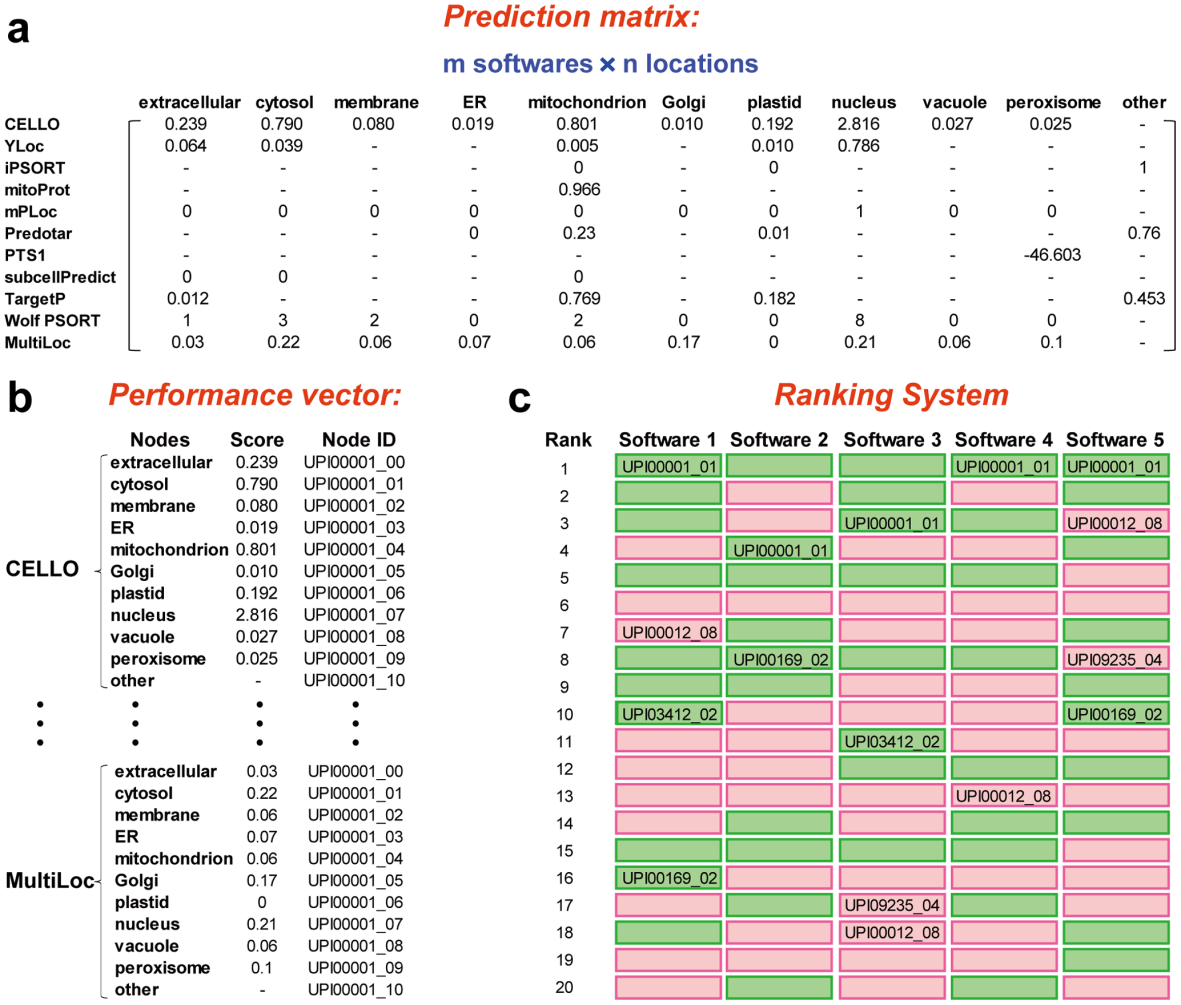
Model for group voting. (a) Raw prediction results from 11 predictors were arranged in a performance matrix for a given protein sequence. For subcellular locations that could not be predicted by a specified predictor, a short line presented the absence. (b) Performance vector of predictors was obtained from the performance matrices of different sequences. Each node corresponds to a subcellular location with a score from predictor and an id consisting of protein id and subcellular location code. (c) Ranking system was established according to performance vectors. For each predictor, a rank list was given according to the scores ranging from high to low in performance vectors.

For each selected predictor, both single subcellular location performance and overall performance was evaluated by splitting prediction results into nodes with labels of each subcellular location and corresponding confidence score. According to confidence score a ranked list was given. Note that a ranked list for a predictor here contained all prediction nodes given by that predictor to avoid bias. The workflow from the prediction matrix to the ranked list for one sequence is illustrated in Figure 4. For a given protein sequence, raw prediction results from 11 predictors were arranged in a performance matrix (Figure 4a). The performance vector was obtained by splitting prediction results of each predictor into ten nodes with sub-location information and a corresponding confidence score (Figure 4b). The ranking system was established according to performance vectors. For each predictor, a rank list was given according to the scores ranging from high to low in performance vectors (Figure 4c).

Assessments for each subcellular localization predictor are given by area under the ROC curve (AUROC, true positive rate vs. false positive rate) [32], the area under the precision vs. recall curve (AUPR) [32], F-score [33] and Matthews Correlation Coefficient (MCC) [33]. True positive rate (TPR) and false positive rate (FPR) as a function [9] of cutoff-value k were defined as follows [32]:
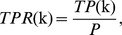


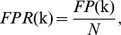


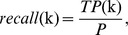


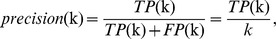


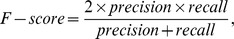
where TP(k) is the number of true positive edges and FP(k) is the number of false positive nodes in top k predictions in the ranked list, while P is the number of total positive nodes and N is the number of total negative nodes according to the gold standard.

Since subcellular localizations of proteins not verified should be considered as potentially existent rather than non-existent, only true positive and false positive rates can be confirmed. Furthermore, by defining arbitrarily which of the two features is positive or negative, intersection *k* of recall(k) curve and precision(k) curve was calculated for each prediction method using the ranked list and gold standards. Consequently, the top k nodes were defined as positive predictions and others were regarded as negative predictions. The following four quantities were defined: true positives (tp) = number of positive events that are correctly predicted; true negatives (tn) = number of negative event that are correctly predicted; false positives (fp) = number of negative events that are incorrectly predicted to be positive; and false negative (fn) = number of subjects that are predicted to be negative despite they are positive. Finally, the MCC was defined as [33]:
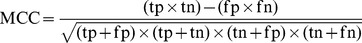



### Singular value decomposition analysis

Before the integration of predictors, singular value decomposition (SVD) analysis was performed to portray similarities and variances of the predictors. Feature vectors were derived directly from each predictor's ranked list to form a performance matrix. Feature vectors consist of ranks for individuals where smaller ranks stand for higher confidence scores. Since subcellular locations predictable from different predictor varied, feature vectors of same dimensions were put in one matrix for SVD via SVDLIBC. Default parameters were used to perform this task.

### Integration of predictors by group-voting

According to the ranked list used in the assessment procedure, a normalized score of each individual node was introduced via calculating the average value of all predictors [28]. Note that some predictors capable of predicting only several locations gave nodes with label ‘others’ and ‘no found’ which were not taken into account for further analysis.

To balance the different predictable locations of predictors that have different length of ranking range, the percentage (rank divided by rank range) rather than rank was calculated. The normalized score was given by
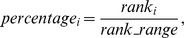


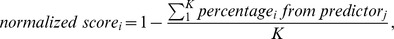
where rank_i_ was calculated from the above method for node_i_, rank range is the largest rank the predictor could give, K is the number of predictors that was able to give corresponding predictions for node_i_. Some statistical tests were then performed to examine whether the difference between true positive ranks and false positive ranks was significant. Finally, the group voting performance was estimated by the AUROC value.

### Integration of predictors by artificial neural-network

The artificial neural-network took the raw score as the input from predictors for each protein sequence. The inputs function and activate function were chosen as:
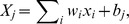


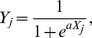
where w_i_ is the weight, b denotes bias, and with the activate function as a logsig function. Topological structure was determined and stepwise-selection of predictors was performed to form the best community for prediction. The predictor's results were added one by one to form input feature subsets ordered according to their accuracy (AUROC value) from high to low. Whenever a predictor's come-in caused a decline in AUROC, the predictor's results were abandoned. Then the optimal combination of predictors was tested in different network structure to achieve the best prediction results.

### P-values as statistical inference for significance

Random sequences of amino acid residues randomly ranging from 50 to 300 were generated through a simple perl script. These sequences were considered to be negative sample which were non-existent in any of the ten subcellular locations. The negative sample was utilized for P-value construction, which will be further illustrated in “P-values as statistical inference for significance”. BLASTP searches for the generated random sequences were performed against the whole proteomes of Arabidopsis and maize. Hits with an identity larger than 30% were also removed to avoid bias. Sequences generated in such methods were considered non-existent and prediction results through the meta-predictor constituted the null hypothesis, from which the P-value was drawn. Note that the P-value constructed in this way reflected the confidence that the sequence was existent in certain cellular compartment according to the null hypothesis. Here smaller prediction scores corresponded to higher P-values, meaning that the predictor had a higher confidence that the protein was non-existent in that subcellular location.

## Supporting Information

Figure S1 **Frequency-distribution for TP nodes vs. FP nodes of single best predictor (cello), integration of 3 predictors (cello, Wolf PSORT, MultiLoc) and integration of 11 predictors were shown with AUPR and AUROC values.** Significant difference between distribution means could be seen. AUPR and AUROC values raised as number of predictors integrated increased.
(TIF)

Table S1 **A detailed summary of currently available predictors.**
(XLSX)

Table S2 **ANOVA for the performance of individual predictors with PSI.**
(XLSX)

Table S3 **Ranking results and normalized scores for individual predictors.**
(ZIP)

Table S4 **Performance comparison between PSI and other individual predictors.**
(XLSX)

Table S5 **Predicted results for the proteins in other plants by PSI.**
(XLSX)
